# Supplementary material for: Homological landscape of human brain functional sub-circuits
Source: Mathematics (Basel). Author manuscript; Available in PMC 2025 Aug 15. (PMC12352577; doi:10.3390/math12030455)
Supplement: Supplementary Material [file NIHMS2047931-supplement-Supplementary_Material.pdf]

Homological landscape of human brain functional sub-circuits supplementary material

Duy Duong-Tran [1,2,†], Ralph Kaufmann [3,†], Jiong Chen [1,4,†], Xuan Wang [5], Sumita Garai [1], Frederick H. Xu [1], Jingxuan Bao [1], Enrico Amico [6,7], Alan David Kaplan [8], Giovanni Petri [9,10,11], Joaquin Goni [12,13,14], Yize Zhao [15], Li Shen [1*]

1 Department of Biostatistics, Epidemiology and Informatics, Perelman School of Medicine, University of Pennsylvania, PA 19104, USA
2 Department of Mathematics, United States Naval Academy, Annapolis, MD 21402, USA
3 Department of Mathematics, Purdue University, West Lafayette, IN 47907, USA
4 Department of Bioengineering, School of Engineering and Applied Science, University of Pennsylvania, PA 19104, USA
5 Department of Electrical and Computer Engineering, George Mason University, Fairfax, VA 22030, USA
6 Neuro-X Institute, Swiss Federal Institute of Technology Lausanne, Geneva 1015, Switzerland
7 Department of Radiology and Medical Informatics, University of Geneva, Geneva 1211, Switzerland
8 Computational Engineering Division, Lawrence Livermore National Laboratory, Livermore, CA 94550, USA
9 CENTAI Institute, 10138 Torino, Italy
10 NPLab, Network Science Institute, Northeastern University London, London, E1W 1LP, United Kingdom
11 Networks Unit, IMT Lucca Institute, 55100 Lucca, Italy
12 Purdue Institute for Integrative Neuroscience, Purdue University, West Lafayette, IN 47907, USA
13 School of Industrial Engineering, Purdue University, West Lafayette, IN 47907, USA
14 Weldon School of Biomedical Engineering, Purdue University, West Lafayette, IN 47907, US
15 School of Public Health, Yale University, New Heaven, CT 06520, USA

* Corresponding email: `li.shen@pennmedicine.upenn.edu`
† These authors contributed equally to this work.

Persistent diagram

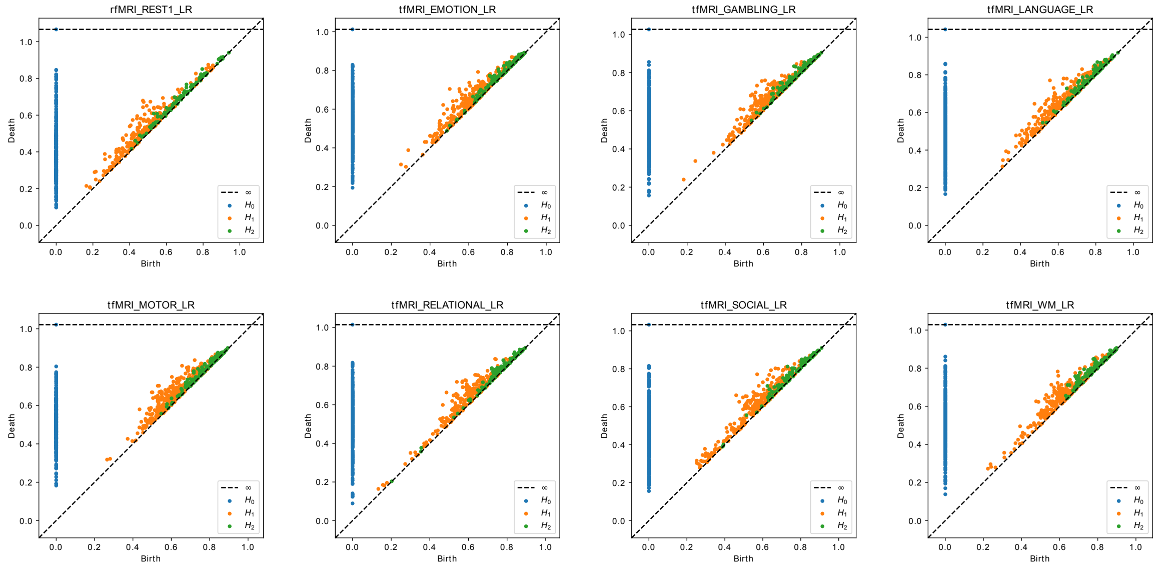

Figure S1. Persistent diagram of the global, group-averaged connectome. Figures shown above are the persistent diagram for all 8 tasks in 3 homological groups, represented by blue, orange, and green dots respectively. The connectome is obtained by taking the average of 100 unrelated HCP subjects' functional connectome.

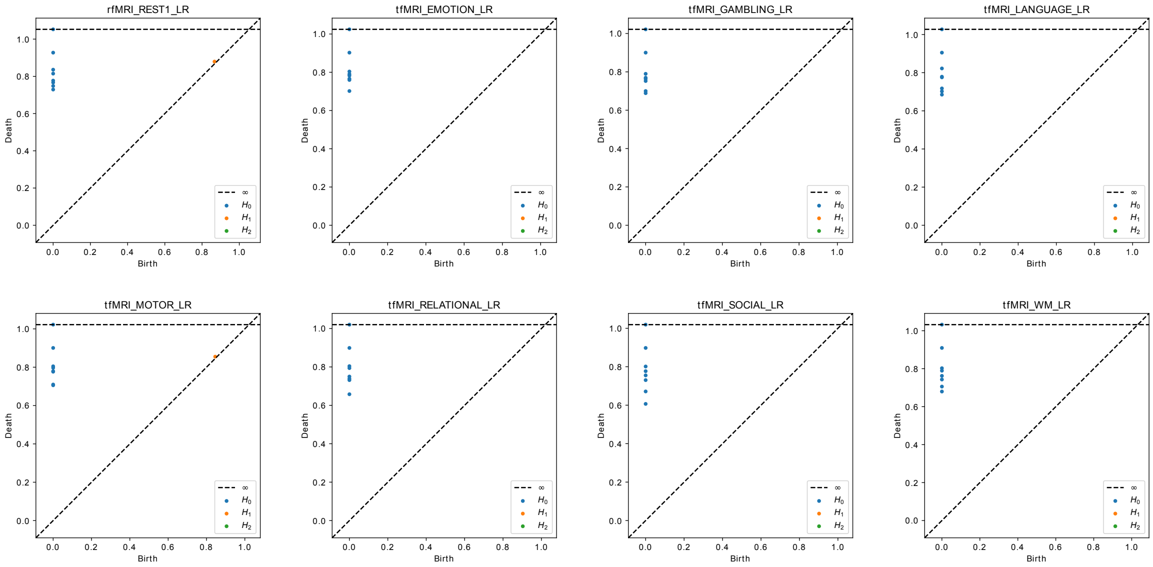

Figure S2. Persistent diagram of the consolidated, group-averaged connectome. Figures shown above are the persistent diagram for all 8 tasks in 3 homological groups. The connectome is obtained by consolidating the communities in the global connectome by Yeo functional networks. Since the consolidated graph only has 8 nodes, there are only two homological groups exist (the zeroth homology represented by blue dots and the first homology represented by orange dots).

Group-mesoscopic level analysis for other tasks

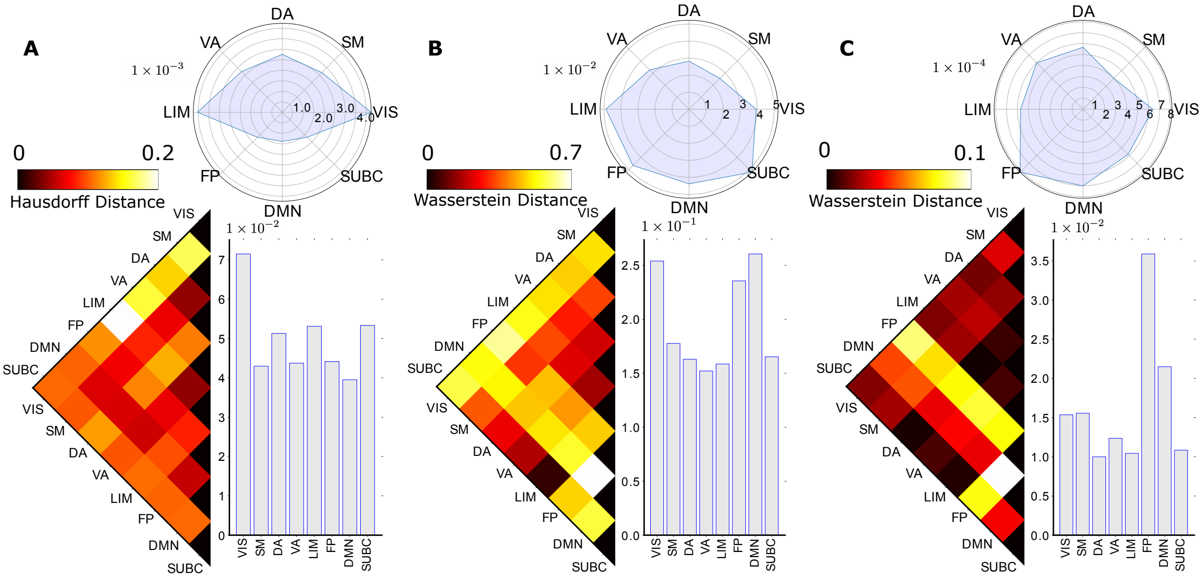

Figure S3. Group-Average Homological distances between brain circuits (FNs) in emotion task. In emotion task, the limbic-visual network pair is the most differentiated FN pair in zeroth homology, while the frontoparietal network and default mode network are the most distinct pair of FNs. The visual network and frontoparietal network have the highest average distance compared with other FNs in the zeroth and second homology, but in the first homology, the visual network, frontoparietal network, and default mode network all have high average distance. The limbic and visual networks also have high variance in distance compared to others.

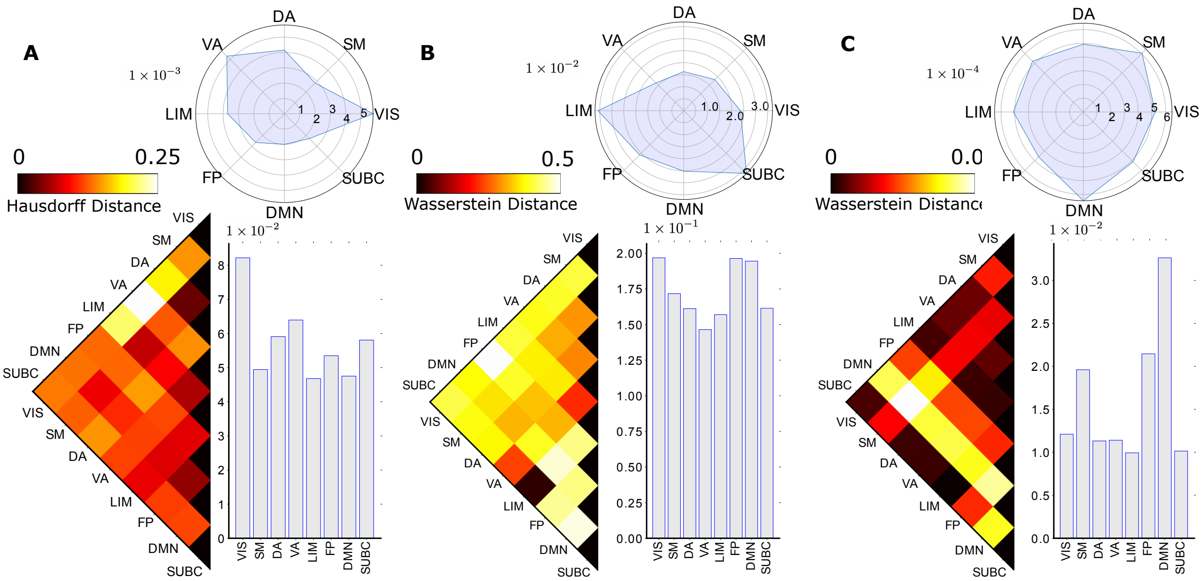

Figure S4. Group-Average Homological distances between brain circuits (FNs) in gambling task. In gambling task, the visual network with ventral attention pair, visual network with frontoparietal network pair, and somatomotor network with default mode network pair are the most differentiated FN pairs in the zeroth, first, and second homological group respectively. The visual and default mode networks have the highest average distance in the zeroth and second homology, while in the first homology, the visual network, frontoparietal, and default mode network all have high average distance. The ventral attention and visual networks have high variance in zeroth homology, and the limbic and subcortical systems have high variance in the first homology.

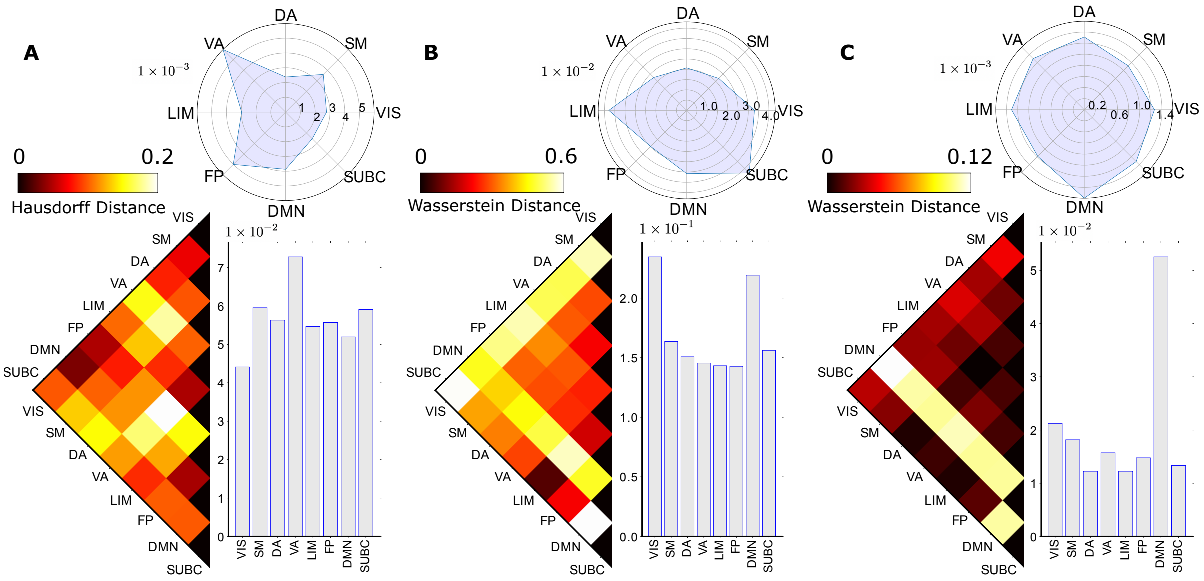

Figure S5. Group-Average Homological distances between brain circuits (FNs) in language task. In language task, the frontoparietal with ventral attention networks are the most distinct pair of tasks in the zeroth homology. In the first homology, the subcortical system with both visual and default mode networks all have a high Wasserstein distance. In addition, the default mode with visual networks is the most distinct pair of tasks in the second homology. The ventral attention, visual, and default mode networks have the highest average distance in the zeroth, first, and second homological groups, with ventral attention, subcortical, and default mode systems having the highest variance. The default mode also serves as the clear winner in the second homology in distance heatmap as well as average distance, indicating its high involvement in the language task.

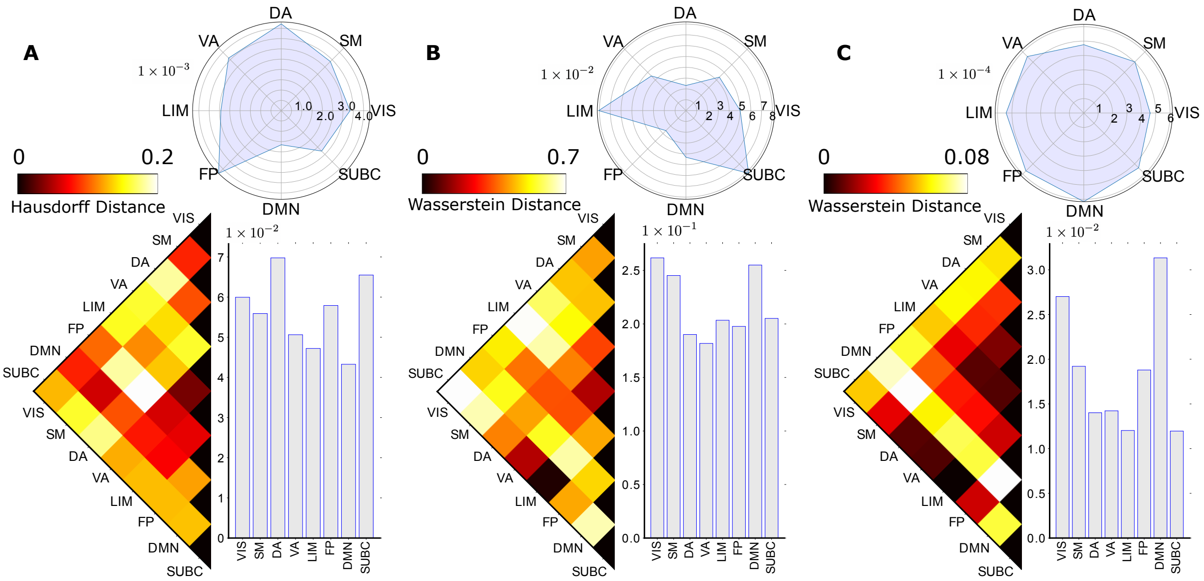

Figure S6. Group-Average Homological distances between brain circuits (FNs) in motion task. In motion task, the frontoparietal with dorsal attention networks are the most distinct pair of tasks in the zeroth homoloyg. In the first homology, the visual network with both subcortical and limbic system all have a high Wasserstein distance. In the second homology, the default mode with both somatomotor and frontoparietal networks have a high Wasserstein distance. The dorsal attention, visual, and default mode networks have the highest average distance in the zeroth, first and second homological groups. The visual network and frontoparietal networks have the highest variance in the zeroth and second homology, while in the first homology, the default mode network and subcortical system both have a high variance.

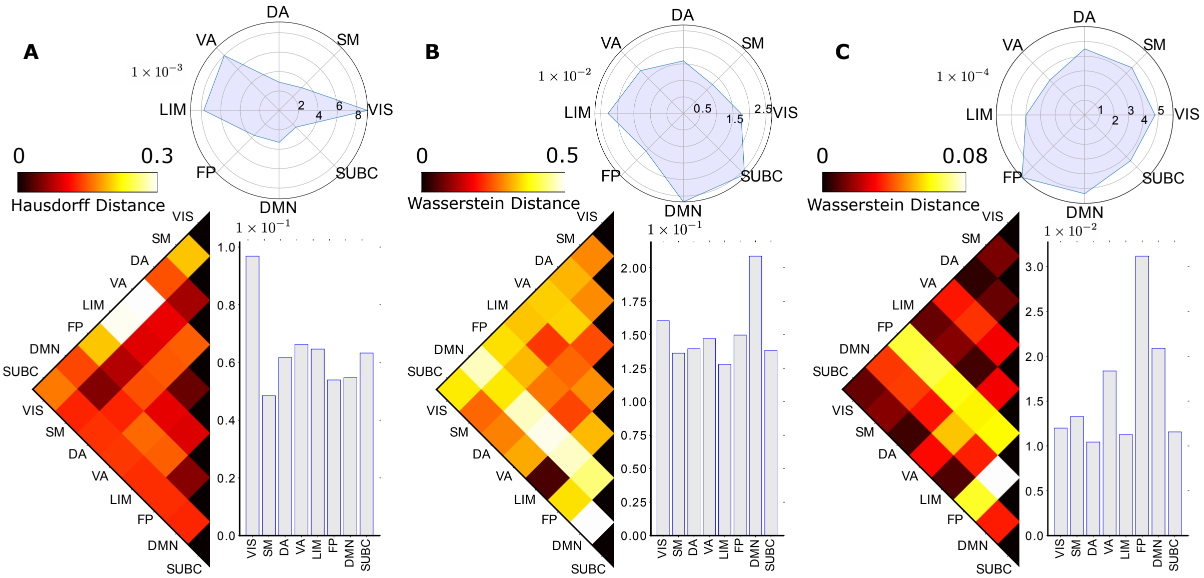

Figure S7. Group-Average Homological distances between brain circuits (FNs) in relational task. In relational task, the visual network with both limbic and ventral attention networks have the highest Hausdorff distance. The visual network with ventral attention also has the highest Wasserstein distance in the first homology, and in the second homology, the frontoparietal and default mode network pair has the highest distance. The relational task is also consistent in the average distance with the zeroth and first homology, having the visual network as the most distinct network. The frontoparietal network has the highest average distance in the second homology. In all homological groups, the visual network has very high variance, and the subcortical system in H1 and the frontoparietal network in H2 also have high variance compared to others.

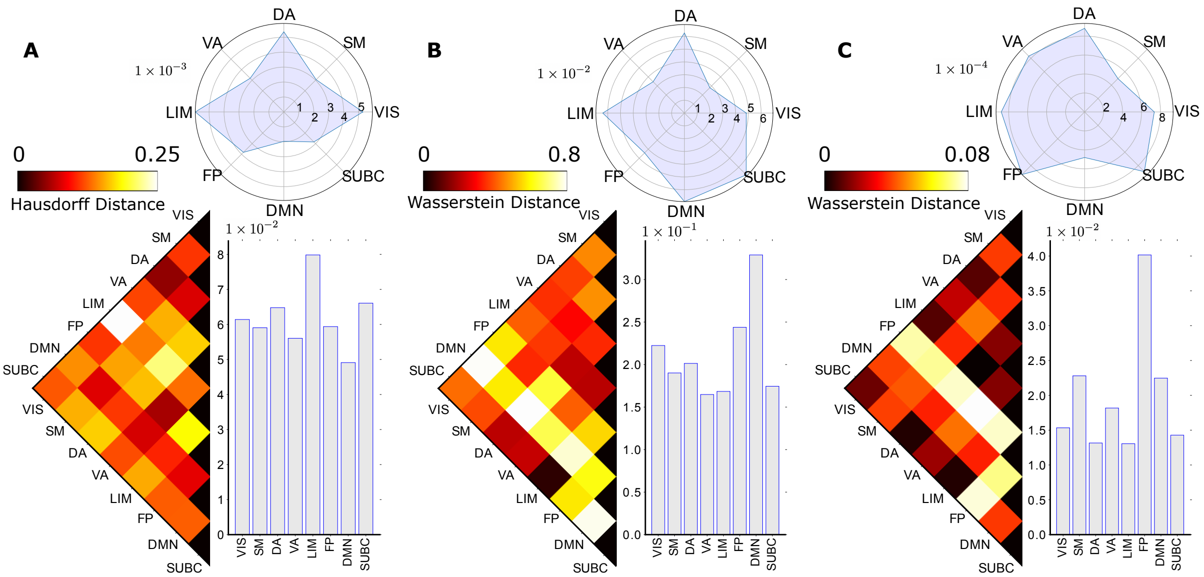

Figure S8. Group-Average Homological distances between brain circuits (FNs) in social task. In social task, the limbic network and visual network pair is the most differentiated FN pair in the zeroth homology. The first homology has multiple distinct FN pairs, and all of them involve the default mode network. A similar pattern is observed in the second homology with the frontoparietal network as the consistent network in those FN pairs. These results also correspond with the average distance, where the limbic network, the default mode network, and the frontoparietal network is the most distinct network in the three homological groups, and at the same time they also have the highest variance.

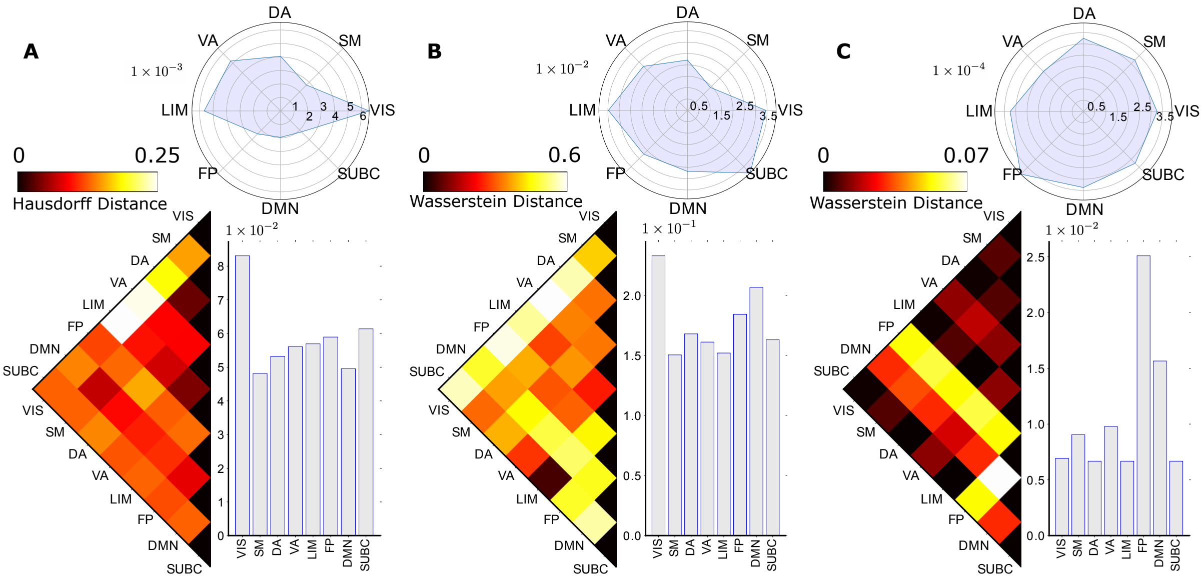

Figure S9. Group-Average Homological distances between brain circuits (FNs) at working memory task. In working memory task, the zeroth homological group has the limbic-visual network pair as the most differentiated pair, and the visual network also has the highest average distance and variance. The ventral attention and visual network pair has the highest distance in the first homology, with the visual network still being the most distinct network, but the subcortical system has the highest variance. In the second homology, the strong separation in the frontoparietal network is consistent with the average distance plot as well as the variance plot that the frontoparietal network is the most differentiated FN, and particularly with the default mode network, the FN pair has the highest Wasserstein distance.

Individual-mesoscopic level analysis

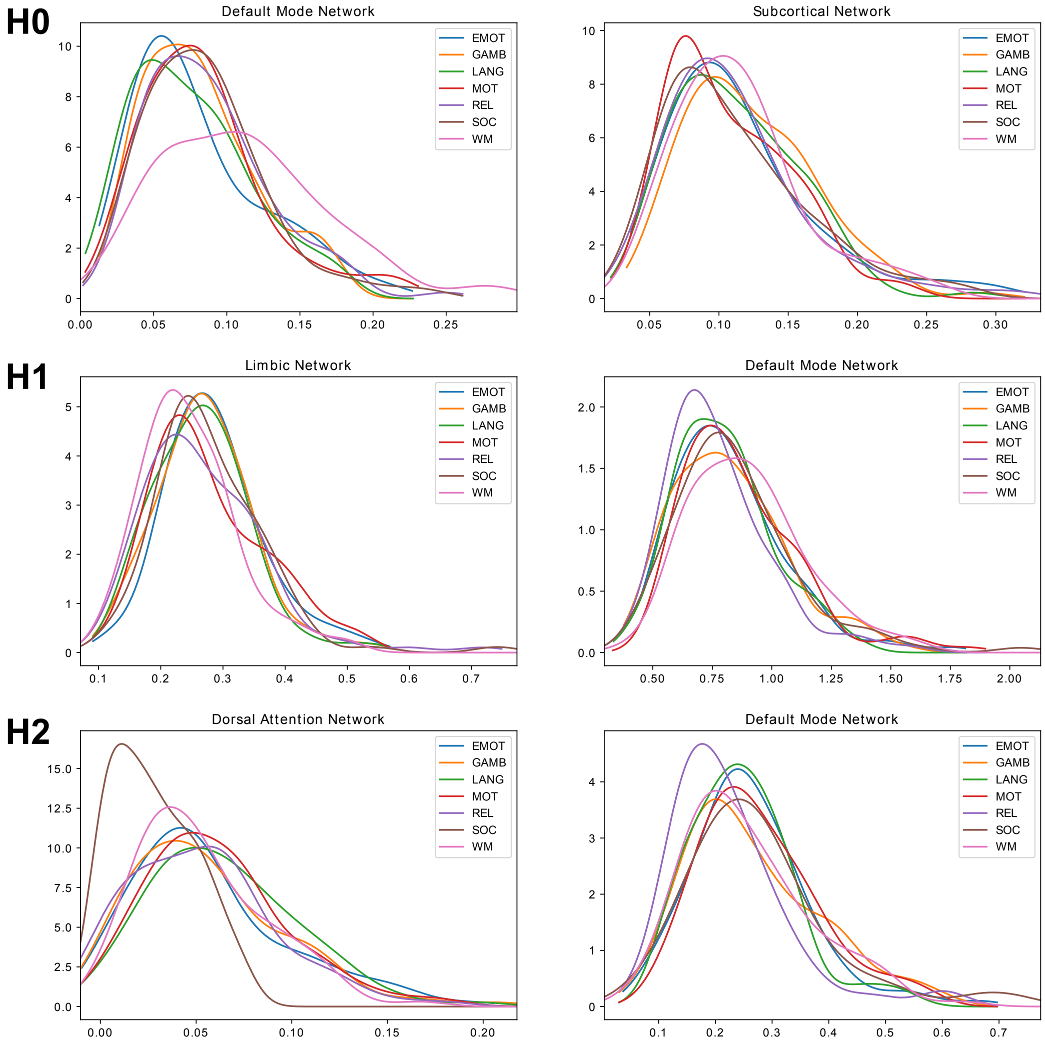

Figure S10. Distribution of pairs of functional networks having the highest distance in three homological groups. The top row (H0), middle row (H1), and bottom row (H2) show the distribution of distance measures between the resting state and the other 7 tasks. Each row involved the FN pair with the highest distance in the group-level mesoscopic analysis at the resting state. The distribution is used to calculate the KL divergence described in the main article.

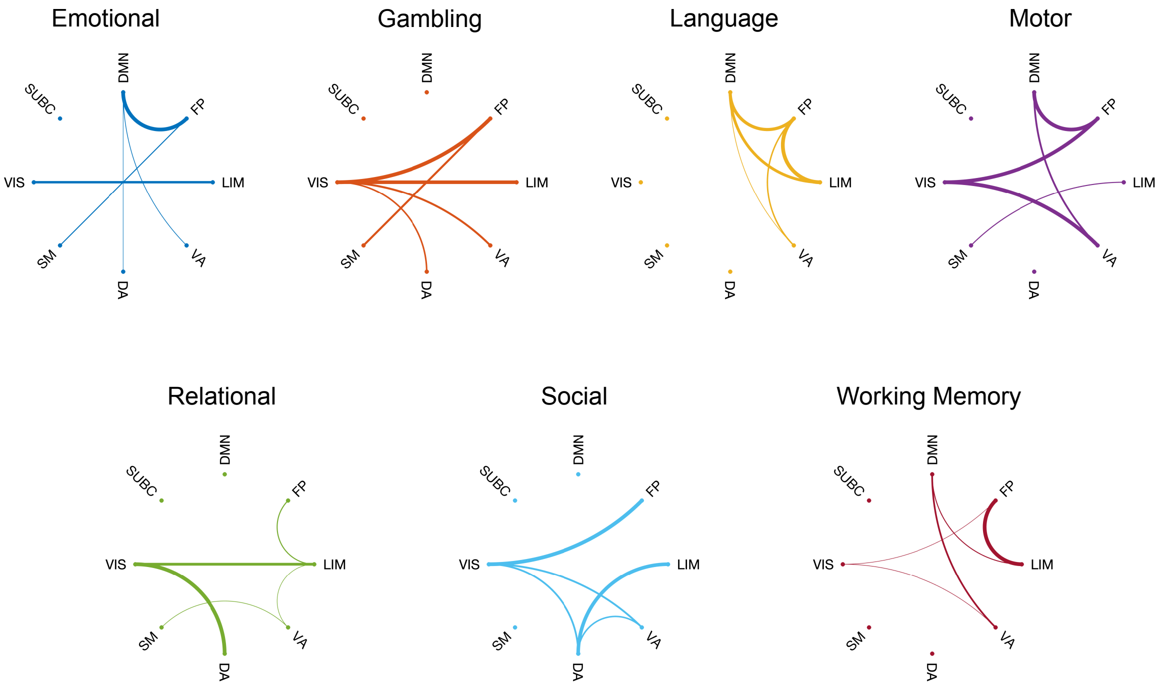

Figure S11. KL divergence circular plot for 7 fMRI tasks-to-REST with functional network comparison in H0. The KL divergence between functional networks (see Figure S10 for example) is visualized with the task-wise circular plot. The top five KL divergence is normalized and demonstrated by the strength of circular connectivity. The pattern in the zeroth homology is more heterogeneous, but we can still identify some hubs such as the visual network in the gambling task.

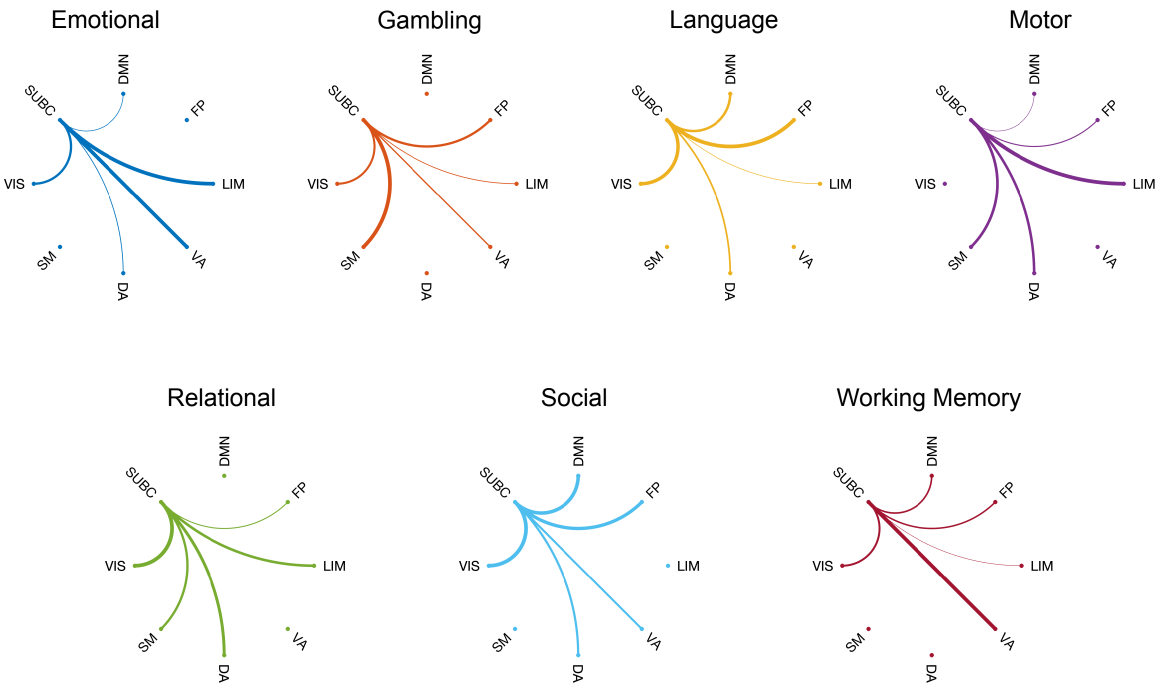

Figure S12. KL divergence circular plot for 7 fMRI tasks-to-REST with functional network comparison in H2. The highest five KL divergence is visualized with the task-wise circular plot and normalized for clearer demonstration. The H2 homology provides a higher level of topological structure view, where we can find that the subcortical system is mostly the hub for all tasks.
